# Evaluation of the Estimation Capability of Response Surface Methodology and Artificial Neural Network for the Optimization of Bacteriocin-Like Inhibitory Substances Production by *Lactococcus lactis* Gh1

**DOI:** 10.3390/microorganisms9030579

**Published:** 2021-03-12

**Authors:** Roslina Jawan, Sahar Abbasiliasi, Joo Shun Tan, Mohd Rizal Kapri, Shuhaimi Mustafa, Murni Halim, Arbakariya B. Ariff

**Affiliations:** 1 Bioprocessing and Biomanufacturing Research Centre, Faculty of Biotechnology and Biomolecular Sciences, Universiti Putra Malaysia, Serdang 43400, Malaysia; roslinaj@ums.edu.my (R.J.); mohdrizal@upm.edu.my (M.R.K.); murnihalim@upm.edu.my (M.H.); 2 Biotechnology Programme, Faculty of Science and Natural Resources, Universiti Malaysia Sabah, Jalan UMS, Kota Kinabalu 88400, Malaysia; 3 Halal Products Research Institute, Universiti Putra Malaysia, Serdang 43400, Malaysia; upmsahar@yahoo.com (S.A.); shuhaimi@upm.edu.my (S.M.); 4 School of Industrial Technology, Universiti Sains Malaysia, Gelugor 11800, Malaysia; jooshun@usm.my; 5 Department of Microbiology, Faculty of Biotechnology and Biomolecular Sciences, Universiti Putra Malaysia, Serdang 43400, Malaysia; 6 Department of Bioprocess Technology, Faculty of Biotechnology and Biomolecular Sciences, Universiti Putra Malaysia, Serdang 43400, Malaysia

**Keywords:** response surface methodology, artificial neural network, optimization, bacteriocin-like inhibitory substances, *Lactococcus lactis* Gh1

## Abstract

Bacteriocin-like inhibitory substances (BLIS) produced by *Lactococcus lactis* Gh1 had shown antimicrobial activity against *Listeria monocytogenes* ATCC 15313. Brain Heart Infusion (BHI) broth is used for the cultivation and enumeration of lactic acid bacteria, but there is a need to improve the current medium composition for enhancement of BLIS production, and one of the approaches is to model the optimization process and identify the most appropriate medium formulation. Response surface methodology (RSM) and artificial neural network (ANN) models were employed in this study. In medium optimization, ANN (R^2^ = 0.98) methodology provided better estimation point and data fitting as compared to RSM (R^2^ = 0.79). In ANN, the optimal medium consisted of 35.38 g/L soytone, 16 g/L fructose, 3.25 g/L sodium chloride (NaCl) and 5.40 g/L disodium phosphate (Na_2_HPO_4_). BLIS production in optimal medium (717.13 ± 0.76 AU/mL) was about 1.40-fold higher than that obtained in nonoptimised (520.56 ± 3.37 AU/mL) medium. BLIS production was further improved by about 1.18 times higher in 2 L stirred tank bioreactor (787.40 ± 1.30 AU/mL) as compared to that obtained in 250 mL shake flask (665.28 ± 14.22 AU/mL) using the optimised medium.

## 1. Introduction

Bacteriocins are defined as peptides or proteins ribosomal synthesized by Gram-positive and Gram-negative bacteria that inhibit or kill other related or unrelated microorganisms [1,2]. Inhibition mechanisms of bacteriocins may have a narrow or broad spectrum by inhibiting taxonomically close or a wide range of bacteria [3]. Although bacteriocins could be produced by several microorganism, bacteriocin produced by lactic acid bacteria (LAB) are of particular interest to the food industry [4,5]. The use of acronym BLIS (bacteriocins-like inhibitory substance) referred to uncharacterised inhibitory agents that appear “bacteriocin-like” in their activity [6]. BLIS is often interchangeably used with bacteriocin since BLIS is uncharacterised bacteriocin that apparently shares similar activity and can be identified by using the same quantification methods as bacteriocin [7].

Recently, bacteriocins have attracted considerable interest for use as safe food preservatives, as bacteriocins are easily digested by the human gastrointestinal tract [3]. The use of bacteriocins as natural food preservatives fulfils consumer demands for high quality and safe foods without the use of chemical preservatives. The application of bacteriocins for biopreservation of foods usually includes the following approaches; (i) inoculation of food with the bacteriocin-producer strain, (ii) addition of purified or semipurified bacteriocin as food additive; and (iii) use of a product previously fermented with a bacteriocin-producing strain as an ingredient in food processing [8,9]. Despite extensive research on the properties and applications of various bacteriocins, to date, only nisin (produced by *Lactoccocus lactis* subsp. *lactis*) and pediocin (produced by *Pediococcus acidilactici*) are used commercially in the food industry [10,11].

The application of bacteriocins as food additives is limited for various reasons, which include the effectiveness of pathogen elimination and high cost of bacteriocins production [9]. As bacteriocin-producing LAB need complex nutrition to grow, this leads to increase in the production cost and also the difficulties for the purification. Medium optimization and formulation are essential for the success of an industrial fermentation as it directly affects the yield, productivity and costs of bioproducts [12].

It is known that bacteriocin production by LAB is affected by culture conditions as well as culture medium composition [13,14]. Optimisation of fermentation factors is critical to maximising the yield of a specific product before large scale production [15]. To observe the effect of medium components, the experiment must be designed so that each medium component is uniformly distributed throughout its sample space [16]. Optimization of media composition has been extensively employed for enhancement of bacteriocin production by LAB as reported previously [17,18,19,20].

Various strategies have been used to optimise the medium formulation for improvement of growth and also the production of the target product. The classical approach, known as one-factor-at-a-time (OFAT), involves laborious experimental set up and time consuming. The modern statistical and mathematical techniques, which include response surface methodology (RSM), artificial neural network (ANN), genetic algorithm (GA) and Nelder Mead (NM) simplex have been applied with a smaller number of experiments as compared to OFAT. Each methodology has advantages and disadvantages, and the specific techniques are applied to achieve the most reliable results. Some desirable results could also be achieved from the use of a combination of various optimisation techniques [12]. The ability of RSM and ANN to assess the interrelationship effects between the influencing factors leads to the better prediction of optimum point as compared to OFAT [21,22].

Therefore, in this study, the experimental designs provided by statistical analysis system were used for the optimisation of fermentation medium for improvement of BLIS production by *Lactococcus lactis* Gh1. The optimised medium was then subjected to subsequent experiment to evaluate the effect of various impeller speeds on growth of *Lactococcus lactis* Gh1 and production of BLIS in 2 L stirred tank bioreactor.

## 2. Materials and Methods

### 2.1. Microorganisms and Fermentation

The method of culture preparation and fermentation conditions were as that described by Jawan et al. [23]. The stock culture of *L. lactis* Gh1 was first revived on the Brain Heart Infusion (BHI) agar (Merck, Darmstadt, Germany) prior to the preparation of inoculum. A single colony of *L. lactis* Gh1 was cultured in 10 mL of BHI broth (Merck, Darmstadt, Germany) and incubated at 30 °C for 24 h. The 1% (*v*/*v*) of the culture was subcultured at 30 °C for 16 to 18 h before being used as an inoculum. The optical density (OD) of the culture at 650 nm was standardized at OD600 of 1.89–2.00 (~2.68 × 10^9^ CFU/mL) and used as an inoculum for all fermentations with the size of 1% (*v*/*v*). All experiments were conducted in 100 mL of BHI broth in 250 mL of Erlenmeyer flasks. The cultures were incubated at 30 °C in a horizontal shaker (B. Braun Biotech International, Melsungen, Germany) and agitated at 100 rpm for 24 h.

### 2.2. One-Factor-at-a-Time (OFAT)

The selection of suitable culture medium, replacements of carbon and nitrogen sources, and preliminary screening of medium components has been performed and published in our previous work [24]. In this study a range of concentrations of modified BHI medium component (soytone, sodium chloride, di-sodium hydrogen phosphate and fructose) were varied as shown in Table 1. During the screening of one factor, the other factors were fixed according to the original composition of the BHI medium. Identification of fermentation performance, variables and their selected level was determined by the production of BLIS. The variables with significant BLIS enhancement were applied in the subsequent experiments.

### 2.3. Response Surface Methodology Modelling (RSM)

The components of medium were used as variables in the optimization strategies. The variables were varied according to the design of experiments set by RSM and ANN. The effects of the variables that yield the maximum production of BLIS were identified. Experimental values from predicted optimal conditions were used as validating set and compared with the predicted optimal values.

A total of 30 experiments were conducted according to Box-Wilson (BW) 2^4^ half factorials central composite design (CCD). Each variable set at five different levels of variation (Table 2). The first 16 experiments (2^4^ = 16, factorial CCD) were at factorial points, 8 at axial points (α = 2), and 6 replications for the central points. In RSM method, second-order model in Equation (1) was used to calculate the predicted response and optimal levels.

Y = χ_0_ + χ_1_β_1_ + χ_2_β_1_^2^+ χ_3_β_1_β_2_ (1)

where Y is the predicted response, β_1_ experimental variables, χ_0_ the offset term, χ_1_ the linear effect, χ_2_ the squared effect and χ_3_ the interaction effect.

Equation (1), a quadratic polynomial equation, was generated using Design Expert version 12 (State-Ease, Minneapolis, MN, USA) to analyse the responses of the BLIS production. The results of the analysis of this design were expressed in terms of polynomial coefficients, and the significance of the model was verified by applying the ANOVA analysis. Additionally, R and R^2^ values were calculated to measure the goodness of fit of this regression model. The regression equation was optimized by an iterative method to obtain optimum values. The relationships between response value and the selected medium were represented in the form of 3D response surface plots. The experimental design used for the study is shown in Table 2.

### 2.4. Artificial Neural Network Modelling

Neural Power version 2.5 (CPC-X Software) is a powerful Artificial Neural Network (ANN) program in multinonlinear regression. It was selected for simulation on similar experimental data set for RSM. In ANN modelling, the CCD experimental data was divided into two sets: training set (27 data) and testing set (6 data) with four input variables and one output response.

All ANN models were trained, and the performance of the networks consulted with the test set during training to avoid over-train by the network and thereby improves the predictive ability of ANN network towards data excluded from the training set. All training and testing processes were performed to obtain minimum root mean square error (RMSE) (Equation (2)), maximum correlation coefficient and coefficient of determination.

The models are able to define the true behaviour of the system when R^2^ (Equation (3)) closes to 1.0 and RMSE and mean absolute error (MAE) (Equation (4)) closes to zero, simultaneously. The evaluation of the output error of ANN between the observed and predicted output values was compared using RMSE and MAE. The coefficient of determination, R^2^ of the regression model between the predicted output values and the observed values were also exploited as a measure of performance of ANN and RSM configurations.

(2) $$\mathrm{RMSE}\;=\;\sqrt{\frac{1}{n}\sum_{i=1}^{n}{\left(x_{d}-x_{p}\right)}^{2}}$$

(3) $$R^{2}\;=\;\frac{\sum i=n\;{\left(x_{d}-x_{p}\right)}^{2}}{\sum i=n\;{\left({\bar{x}}_{d}-x_{p}\right)}^{2}}$$

(4) $$\mathrm{MAE}=\frac{1}{n}\sum_{i=1}^{n}\left|x_{d}-x_{p}\right|$$

where *n* is the number of data set, *χ_d_* the desired (observed) values, *χ_p_* the predicted and *χ_d_* the average desired (observed) values.

### 2.5. Verification of Predicted Data

The estimation capabilities of both RSM and ANN models were evaluated by means of comparing the responses computed from both methods to the observed data. The calculated coefficients of determinations, MAE, RMSE and R^2^, were exploited for the purpose of comparison.

### 2.6. Bioreactor Set up and Fermentation

**Figure 1:**
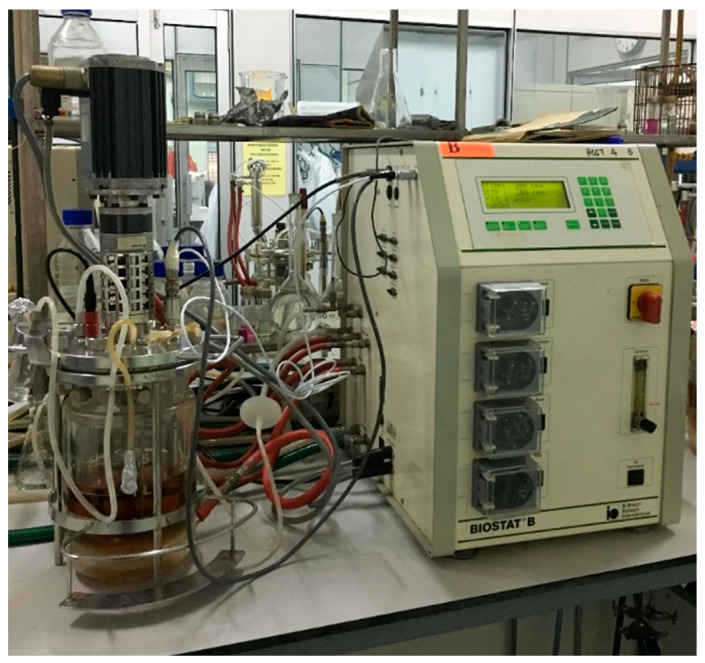
The photograph of 2 L stirred tank bioreactor setting (BIOSTAT, B. Braun Biotech International, Germany) for BLIS production by *L. lactis* Gh1.

**Figure 2:**
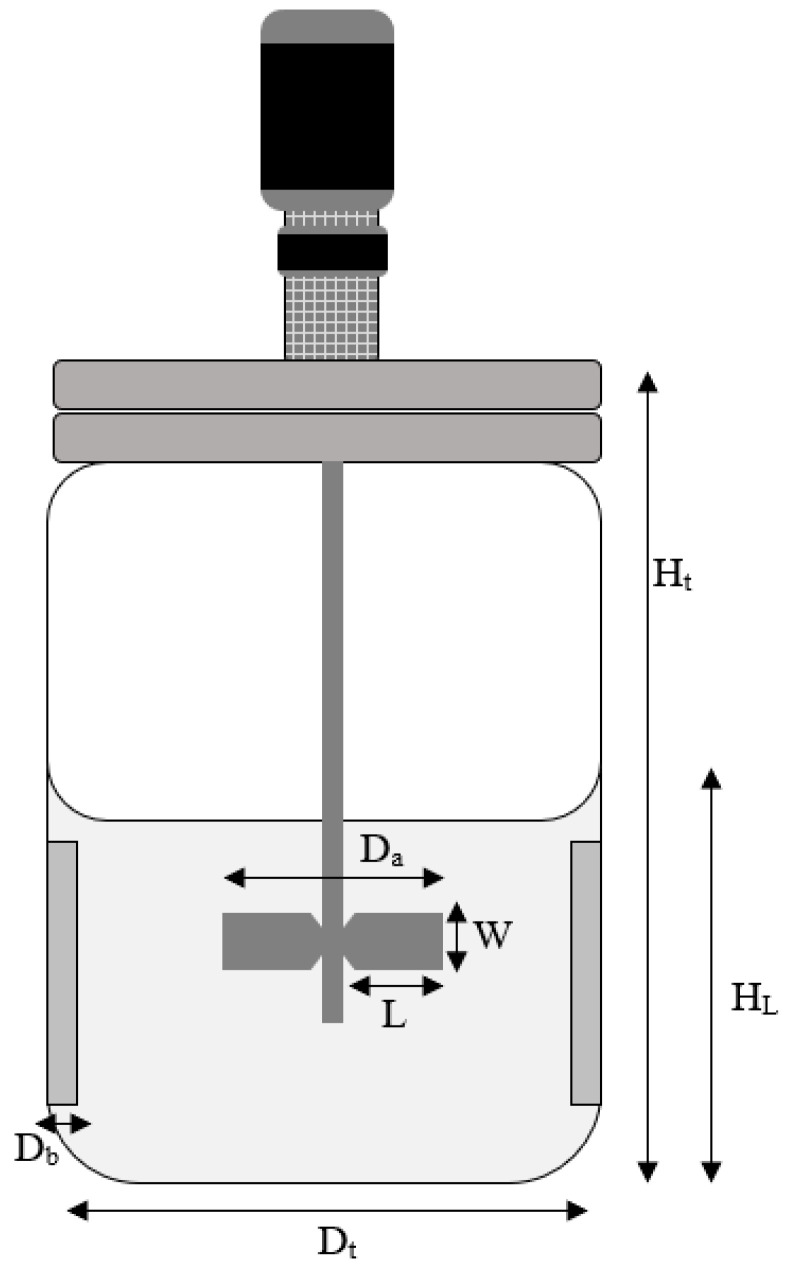
Schematic diagram of 2 L stirred tank bioreactor. Note: H_L_—High of liquid; H_t_—High of bioreactor; D_t_—Diameter of tank; D_a_—Diameter of impeller; D_b_—Diameter of baffles; W—Impeller blade height; L—Impeller blade width.

The performance of *L. lactis* Gh1 for BLIS production using optimized medium, namely FST medium, in stirred tank bioreactor was also evaluated. The cultivation was carried out in a 2 L stirred tank bioreactor (BIOSTAT, B. Braun Biotech International, Germany) with a working volume of 1 L. Configurations and dimensions of the bioreactor are shown in Figure 1 and Figure 2 and Table 3. The bioreactor was equipped with a single six-bladed Rushton turbine for agitation (impeller diameter = 0.053 m) and control module system for temperature, pH, and dissolved oxygen tension (DOT).

As shown in Figure 1, stock culture was added in a 250 mL Erlenmeyer flask containing 100 mL FST medium. The flask was incubated at 30 °C for 5 to 6 h to obtain the culture with a density of ~2.68 × 10^9^ CFU/mL. To initiate the fermentation, the bioreactor containing 1 L of optimized medium was inoculated with 1% (*v*/*v*) inoculum of *L. lactis* Gh1. During the fermentation, the agitation speed was fixed at 100 rpm (without aeration), and the temperature was set at 30 °C. In subsequent experiment, the agitation speed was fixed at the required impeller speed (100, 200, 400, 600 and 800 rpm) without aeration to evaluate the effect of agitation speed on BLIS production. The culture pH was monitored online using in situ sterilizable pH electrode (Mettler Toledo, Greifensee, Switzerland). The DOT level was measured using a polarographic dissolved oxygen (DO) electrode (Mettler Toledo, Greifensee, Switzerland) and recorded throughout the fermentation. Antifoam reagent (Silicon antifoam, Sigma, Burlington, MA, USA) was added to suppress foaming during the fermentation.

### 2.7. Models

Unstructured models, based on Monod and Luedeking–Piret models, were used for modelling the growth of *L. lactis* Gh1, substrate consumption and BLIS formation, respectively (Equations (5)–(7)).

(5) $$\mathrm{Growth}\;\mathrm{rate}\;\frac{\Delta X}{\Delta t}=\mu X$$

(6) $$\mathrm{BLIS}\;\mathrm{production}\;\mathrm{rate}\;\frac{\Delta P}{\Delta t}=qpX$$

(7) $$\mathrm{Substrate}\;\mathrm{consumption}\;\mathrm{rate}\;\frac{\Delta S}{\Delta t}=-qsX$$

where, *X* is cell concentration, *P* is BLIS production, *S* is fructose concentration, *μ* is specific growth rate, *q_P_* is volumetric BLIS production rate and *q_S_* is volumetric fructose uptake rate.

### 2.8. Analytical Procedures

During the fermentation, samples were withdrawn at time intervals of 2 h for analysis. The cell viability was reported as colony forming units (CFU/mL) using spread plate method. Decimal serial dilutions ranging from 10^1^ to 10^9^ of each suspension in 100 mM sodium phosphate buffer (pH 6.5) was spread evenly on the surface of BHI agar plates in triplicates. After incubation at 30 °C for 24 h, the number of viable cells was determined according to Equation (8):

(8) $$\mathrm{CFU}/\mathrm{mL}=\frac{\mathrm{Number}\;\mathrm{of}\;\mathrm{colony}\;x\;\mathrm{dilution}\;\mathrm{factor}}{\mathrm{Volume}\;\mathrm{of}\;\mathrm{sample}\;(\mathrm{in}\;\mathrm{mL})}$$

The changes in culture pH, optical density and antimicrobial activity (AU/mL) against L. *monocytogenes* ATCC 15313 were determined as described by Jawan et al. [24]. The concentration of fructose and lactic acid were determined by using High Performance Liquid Chromatography (HPLC) (Agilent, Australia). The HPLC system was equipped with Aminex ^®^ HPX-87H, 300 mm × 7.8 mm (Bio-Rad, CA, USA) column and UV detector which was read at 210 nm. Sulfuric acid (0.4 mM) was used as a solvent for elution at a flow rate of 0.6 mL/min. Total nitrogen was estimated by Kjeldahl method [25]. All measurements were performed in triplicates.

## 3. Results

### 3.1. Effect of Modified Media Components on BLIS Production

In media formulation for BLIS production, one-factor-at-one-time (OFAT) approach was first applied to screen the suitable concentrations range of modified BHI ingredients before media formulation prediction executed in RSM and ANN (Table 4). BLIS production among the media components and concentrations was not significantly different (*p* > 0.05). However, significant interaction (*p* < 0.001) between the media components and their concentrations was observed for cell growth and BLIS secretion. Production of BLIS was proportionally increased with the increment in fructose and soytone concentration in the media. As for NaCl and Na_2_HPO_4_, both components were required at low and moderate levels, respectively. The highest BLIS production (620.35 ± 1.19 AU/mL) and cell growth (0.69 ± 0.005 g/L) were recorded at 53.07 g/L soytone, 2.0 g/L fructose, 5.00 g/L NaCl and 2.50 g/L NaH_2_PO. This result indicates that BLIS secretion could be enhanced by the manipulation of media components at particular concentrations in the formulation. Accordingly, all components of modified BHI medium were selected for further optimization using RSM and ANN.

### 3.2. Optimization of Fermentation Parameters Using RSM

The matrix of four variables and predicted response (BLIS production) are shown in Table 5. An analysis of variance (ANOVA) of quadratic regression demonstrated that a quadratic model was most suitable to explain the relationship of the variables and response (Table 5). The corresponding second-order response for the production of BLIS (Equation (9)) shows that the coefficient of determination (R^2^ = 0.79), the F-test analysis (F_model_ = 4.17) and probability value (P_model_ > F = 0. 0001), indicating that the model terms are significant and reliable.

Y = + 608.99 − 1.29 ∗ A + 30.57 ∗ B + 6.05 ∗ C − 2.88 ∗ D + 1.84 ∗ A2 + 1.31 ∗ B2 + 19.63 ∗ C2 + 8.87 ∗ D2 − 9.49 ∗ A ∗ B − 6.55 ∗ A ∗ C − 4.04 ∗ A ∗ D − 7.51 ∗ B ∗ C − 16.05 ∗ B ∗ D − 1.64 ∗ C ∗ D(9)

where Y is the BLIS activity; A, B, C and D are the concentration of soytone, fructose, NaCl and Na_2_HPO_4_, respectively.

The models also showed an insignificant lack of fit, as demonstrated by F-value (4.48) and probability value (P_model_ > F = 0.0558). Adequate precision value (7.863) was higher than 4, indicating an adequate signal, and this model can be used to navigate the design space.

**Figure 3:**
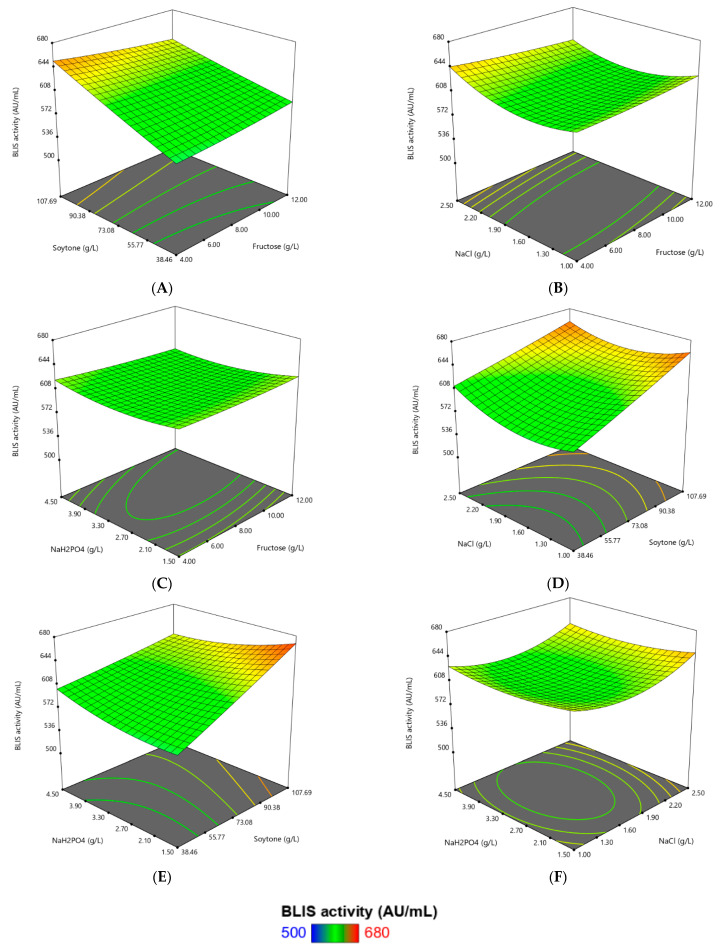
Three-dimensional contour plots of RSM for combined effects of (**A**) Fructose and soytone, (**B**) Fructose and NaCl, (**C**) Fructose and Na_2_HPO_4_, (**D**) Soytone and NaCl, (**E**) Soytone and Na_2_HPO_4_, (**F**) NaCl and Na_2_HPO_4_ on BLIS production by *L. lactis* Gh1.

Figure 3A–F represents the response surface plots intercorrelating three investigated components with BLIS response while the other variables are kept constant at the middle level. The largest coefficient of the amount of soytone indicated that the effect of the amount of soytone was found to be the main influential factor and had a significant and positive impact on the production BLIS. In Figure 3A, BLIS activity increased from 573.63 AU/mL to 653.29 AU/mL and from 589.57 AU/mL to 631.81 AU/mL when the amount of soytone increased from 38.46 g/L to 107.69 g/L at the low and high levels of fructose, respectively. These results showed that BLIS production was increased rapidly with the increasing amount of soytone. A similar trend was also observed in the interactions between soytone and NaCl (Figure 3D) and the interactions between soytone and Na_2_HPO_4_ (Figure 3E).

Meanwhile, the flat and straight slope of response plots in Figure 3C indicated that the concentration of Na_2_HPO_4_ and fructose did not show any specific pattern. At any levels, BLIS activity was not much affected as long as the other fixed variables (soytone and NaCl) added into the medium. NaCl was required at high concentrations for enhancement of BLIS secretion. High BLIS activity was recorded at a high level of NaCl (2.5 g/L) with a low level of fructose (4.0 g/L) and Na_2_HPO_4_ (1.50 g/L), as seen in Figure 3B,F, respectively. BLIS production reduced with decreasing concentration of NaCl from 2.5 g/L to 1.9 g/L and further reduced with a decrease in NaCl to 1.0 g/L.

### 3.3. Optimization of Fermentation Parameters Using ANN

**Figure 4:**
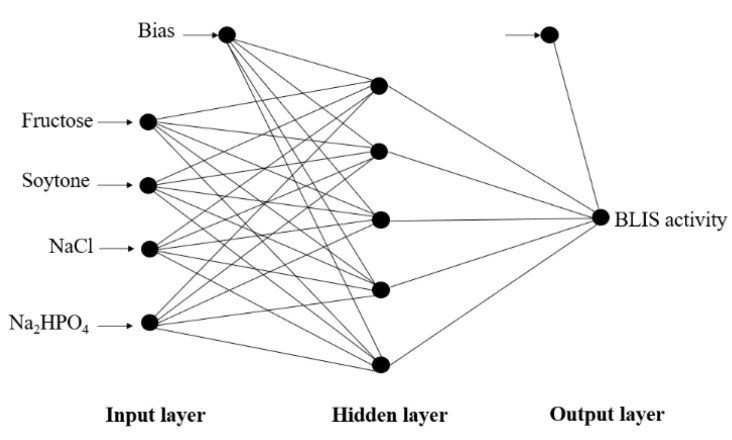
The topology of neural network for the estimation of BLIS production by *L. lactis* Gh1.

**Figure 5:**
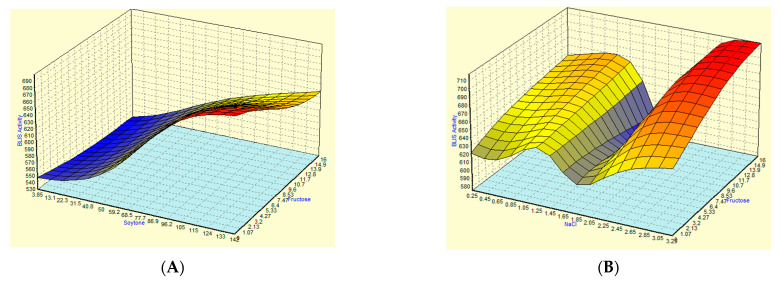

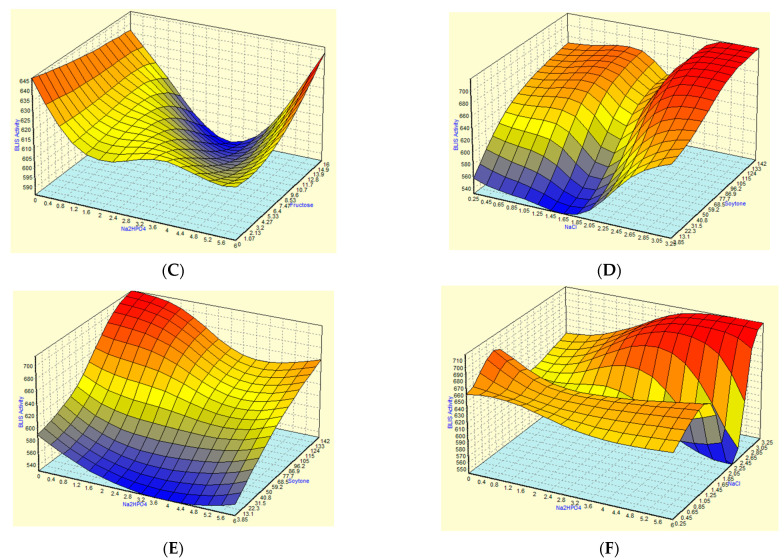
Three-dimensional plots of ANN for the combined effects of (**A**) Fructose and soytone, (**B**) Fructose and NaCl, (**C**) Fructose and Na_2_HPO_4_, (**D**) Soytone and NaCl, (**E**) Soytone and Na_2_HPO_4_, (**F**) NaCl and Na_2_HPO_4_ on BLIS production by *L. lactis* Gh1.

The Quick Prop (QP) was used to predict the BLIS production by *L. lactis* Gh1 on the testing set. The topology of the network consisted of three layers (4-5-1) (Figure 4), an input layer consisting of four fermentation variables, a middle-hidden layer of 5 neurons and one output layer for BLIS activity. The RMSE and determination coefficient of this optimal configuration was 26.02 and 0.97, respectively. Figure 5 shows the three-dimensional plots for the effect of soytone, fructose, NaCl and Na_2_HPO_4_ on BLIS production. These plots present a more flexible and dynamic interaction between the input and output variables as compared to RSM. The interaction between the variables differed as compared to the RSM model. The supplementation of soytone at high concentration (142 g/L) domineered the function of fructose, NaCl and Na_2_HPO_4_ where the highest BLIS activity was achieved with the minimum concentration of these three components (Figure 5A,D,E), respectively. Both fructose (16 g/L) and NaCl (3.25 g/L) were required at maximum concentration to attained high BLIS production (Figure 5B). A similar trend was observed with the interaction between fructose (16 g/L) and Na_2_HPO_4_ (6 g/L) (Figure 5C), while high BLIS secretion was recorded at the maximum concentration of NaCl (3.25 g/L) and Na_2_HPO_4_ (6 g/L) (Figure 5F).

### 3.4. Optimization and Comparison of the Predictive Capability of RSM and ANN Models

The comparison of the yields of BLIS for optimized medium using RSM and ANN is given in Table 6. In RSM, the final predicted optimal values of four variables were simulated, and the values were found to be: 107.63 g/L soytone, 4 g/L fructose, 2.50 g/L NaCl and 1.50 g/L Na_2_HPO_4_. The predicted maximum production of BLIS was 711.14 AU/mL. In ANN, the maximum production of BLIS (717.91 AU/mL) was predicted at 35.38 g/L soytone, 16 g/L fructose, 3.25 g/L NaCl and 5.40 g/L Na_2_HPO_4_. The validation of the model was done by conducting the experiments under the optimal level of fermentation medium. The experimental verification results indicate that ANN (0.1% diff) methodology is superior to RSM (2.13% diff) for the prediction of experimental data with optimum yield of 717.13 ± 0.76 AU/mL and 695.96 ± 2.48 AU/mL, respectively. The BLIS production in optimal medium obtained from RSM and ANN was increased by 1.34-fold and 1.40-fold than that obtained in nonoptimal medium (520.56 ± 3.37 AU/mL), respectively.

The predictive ability of the model was evaluated based on MAE, RMSE, and R^2^ (Table 6). ANN model presents lower MAE (2.20), and RMSE (26.02) values with a determination coefficient close to 1 (0.98) indicate that ANN model has higher predictive ability and accuracy as compared to RSM. On the other hand, RSM model prediction shows higher MAE (15.44) and RMSE (27.08) values with R^2^ far from 1 (0.79). ANN can stimulate the nonlinear system, while RSM is limited to the second-order polynomial system. These results demonstrated that the predictive capability of ANN model was much better than RSM model. Thus, the ANN model is more suitable to be used to describe the interaction between the inputs and output in the production of BLIS by *L. lactis* Gh1.

### 3.5. Growth of L. lactis Gh1 and BLIS Production in the Optimized Medium Using 2 L Stirred Tank Bioreactor

**Figure 6:**
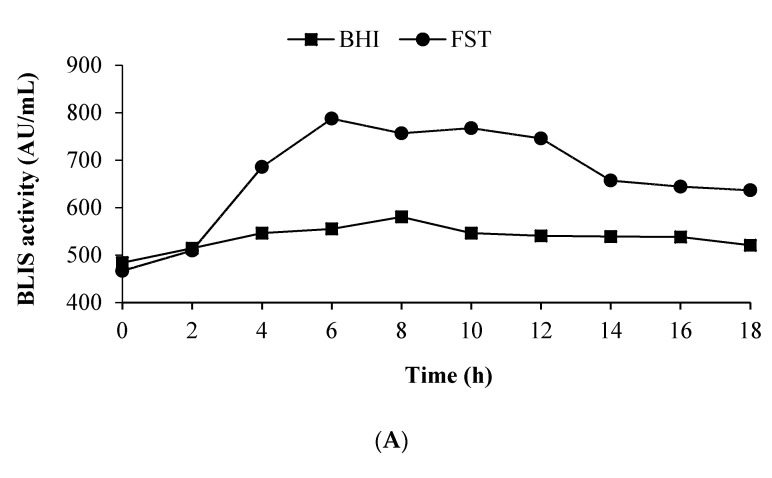

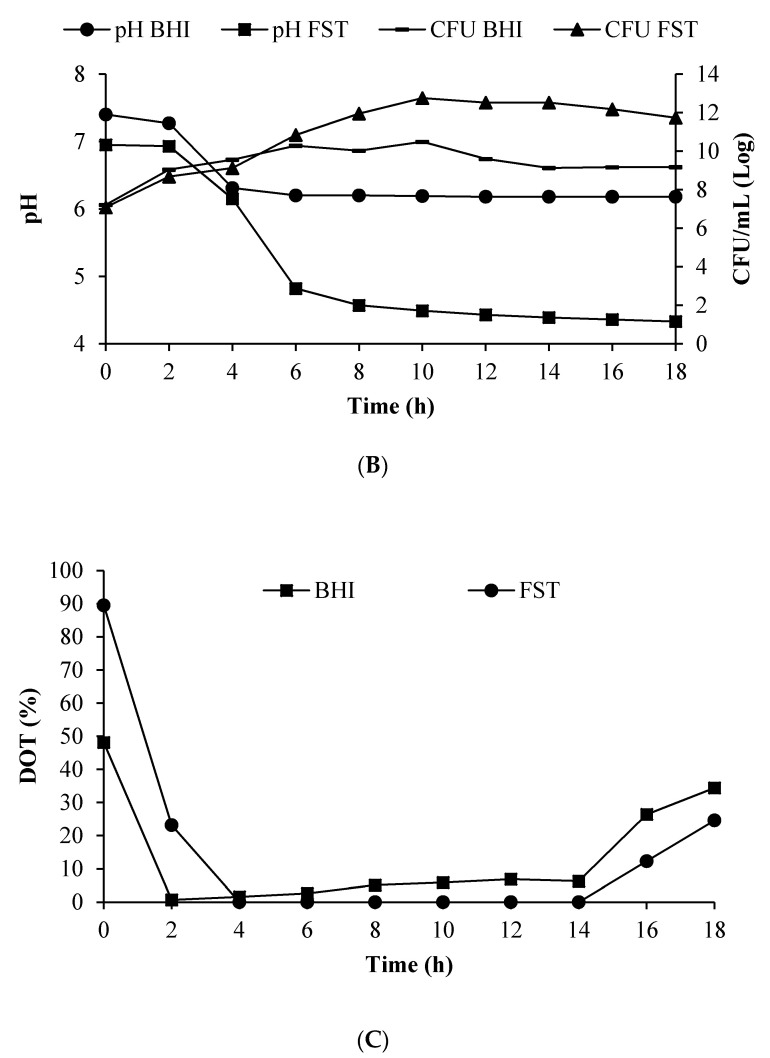
Comparison of the performance of BLIS production by *L. lactis* Gh1 using optimised (FST) and nonoptimised (BHI) media in 2 L stirred tank bioreactor. (**A**) pH reduction and cell viability; (**B**) BLIS activity; (**C**) DOT profile.

The fermentation performance of BLIS production by *L. lactis* Gh1 in both commercial BHI and the optimized (after this referred to as FST) media as predicted by ANN using 2 L stirred tank bioreactor are shown in Table 7 and Figure 6. The maximum BLIS activity (787.40 ± 1.30 AU/mL) and cell concentration (0.87 ± 0.00 g/L) were about 1.36 and 2.02 times higher in the FST medium as compared to BHI medium, respectively (Figure 6A). The fermentation time for FST medium (6 h) to achieved maximum BLIS activity was also shorter than that obtained in fermentation with BHI medium (8 h).

Time to achieved maximum cell concentration was similar in both media (10 h) (Figure 6B). However, the FST (12.76 log CFU/mL) recorded 1.22 higher in cell growth as compared to the BHI medium (10.48 log CFU/mL). Cell reduction was observed in the BHI medium (at h-12), while the cells were maintained in the FST medium with lower pH reduction in line with cell growth. At BHI medium the DOT level was dropped to 0% saturation at h-2 and gradually increased at h-4. While, in FST medium the DOT level dropped to 0% at h-2 and remained for 12 h, before resumed at 16 h of fermentation (Figure 6C).

The BLIS production was improved by scaling up the fermentation scale from shake flask to 2 L stirred tank bioreactor (Table 7). The maximum BLIS activity using FST medium in 2 L stirred tank bioreactor (787.40 ± 1.30 AU/mL) was about 1.18 times higher as compared to FST medium in shake flask (665.28 ± 14.22 AU/mL) at shorter fermentation time (6 h). There was not much difference in maximum cell concentration and specific growth rate in between the shake flask to 2 L stirred tank bioreactor.

### 3.6. Effect of Impeller Speed on BLIS Production by L. lactis Gh1 in Optimised Medium Using 2 L Stirred Tank Bioreactor

**Figure 7:**
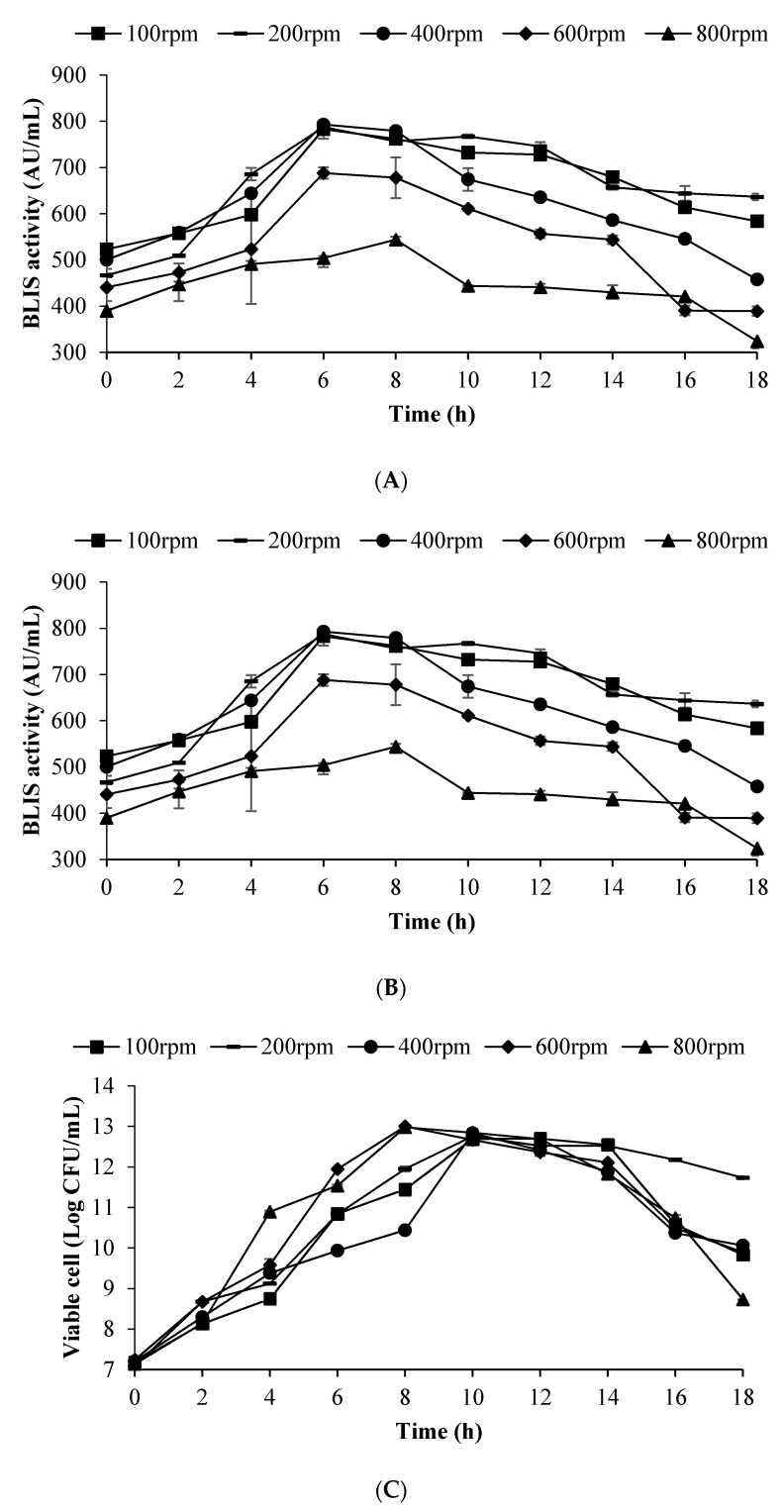

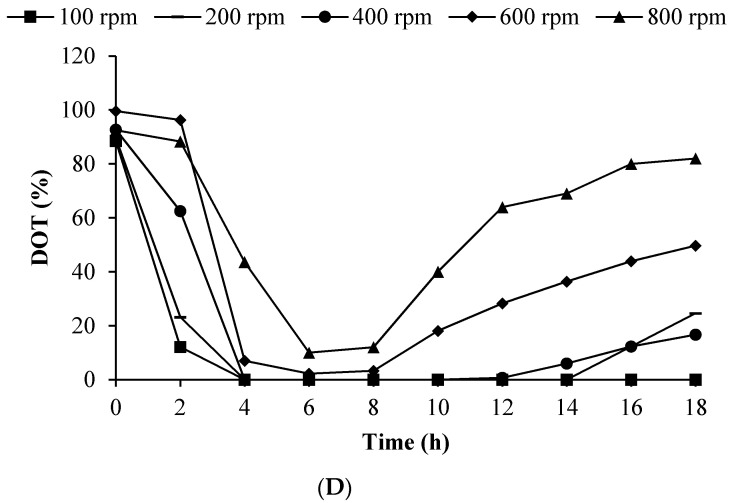
Effect of impeller speed on BLIS activity, cell viability, pH changes and DOT profile during batch fermentation of BLIS by *L. lactis* Gh1 in 2 L stirred tank bioreactor. (**A**) BLIS activity; (**B**) Viable cell; (**C**) pH; (**D**) DOT.

**Figure 8:**
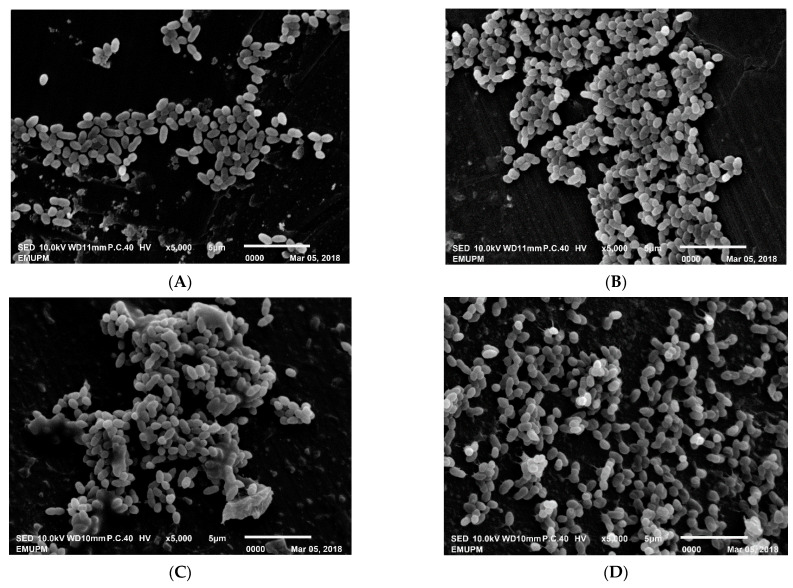

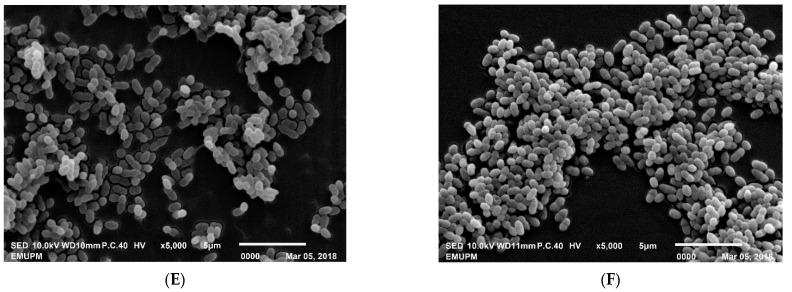
Scanning electron microscopy of *L. lactis* Gh1 at magnification ×5000. (**A**) Control-fresh cells; (**B**) 100 rpm (10-h); (**C**) 200 rpm (10-h); (**D**) 400 rpm (10-h); (**E**) 600 rpm (8-h); (**F**) 800 rpm (8-h).

The performance and the kinetics parameter values for batch fermentation of *L. lactis* Gh1 for the production of BLIS using optimised medium in 2 L stirred tank bioreactor at different impeller speeds are shown in Table 8 and Figure 7 and Figure 8. The production of BLIS was increased slightly with increasing speed of the impeller up to 400 rpm. Reduced in BLIS production was observed at higher impeller speeds (600 to 800 rpm). Among the impeller speed tested in this study, the highest BLIS production (792.91 ± 3.90 AU/mL) was recorded at 400 rpm while the lowest BLIS activity was observed at 800 rpm (543.76 ± 6.83 AU/mL). The lowest cell productivity (Y_BLIS/X_) (523.02 ± 6.57 AU/g), and BLIS production rate (q_p_) (17.74 ± 13.42 AU/g/h) were also observed at 800 rpm. The highest BLIS production rate (q_p_) (287.24 ± 7.98 AU/g/h) and cell productivity, Y_BLIS/X_ (966.21 ± 1.59 AU/g) were recorded at 100 and 200 rpm, respectively.

The highest cell concentration (X_mX_) (1.12 ± 0.002 g/L), total nitrogen consumed (S_mX_) (0.35 ± 0.07 g/L), cell mass yield (0.36 ± 0.00 g cells/g nitrogen) and lactic acid formation (LA_mX_) (2.59 ± 0.15 g/L) were observed at 800 rpm. While, the lowest cell concentration (X_mX_) (0.87 ± 0.002 g/L), total fructose consumed (S_mX_) (7.53 ± 0.04 g/L), cell mass yield (0.10 ± 0.00 g cells/g fructose), lactic acid formation (LA_mX_) (2.13 ± 0.11 g/L), and (µ_mX_) (0.17 ± 0.002 h^−1^) were recorded at 200 rpm. The highest total fructose consumed (S_mX_) (11.95 ± 0.07 g/L) and fructose consumption rate (q_s_) (0.58 ± 0.05 a g/g/h) were observed at 600 rpm, while the BLIS yield (Y_BLISS/S_) (87.54 ± 7.42 AU/g fructose) and cells mass yield (Y_X/S_) (0.13 ± 0.00 g cells/g fructose) were observed at 400 rpm. The maximum specific growth rate (µ_mX_) (0.34 ± 0.008 h^−1^), and BLIS production rate (q_p_) (287.24 ± 7.98 AU/g/h) were observed at 100 rpm.

The cell viability was preserved at 200 rpm, where the highest viable cell concentration (11.73 log CFU/mL) was obtained at 18 h of fermentation. On the other hand, the lowest viable cell concentration (8.73 log CFU/mL) was recorded at impeller speed of 800 rpm (Figure 6B). The high and low cell viability was very much related to the DOT profile recorded at 200 and 800 rpm. At impeller speed of 100, 200 and 400 rpm, the DOT level was dropped to very low levels after 4 h of fermentation and remained at 0% saturation for 18, 10 and 8 h, respectively. In the case of 600 (2.2–3.3%) and 800 (10–12%) rpm, the DOT level was dropped to the lowest for only 2 h. Effect of different impeller speeds (100–800 rpm) on BLIS production by *L. lactis* Gh1is summarised in Figure 7.

The size of the cells was influenced by the variation in impeller speed. The cell size was increased from 0.91 ± 0.07 µm (length) × 0.54 ± 0.06 µm (width) at impeller speed of 100 rpm to 1.26 ± 0.05 µm (length) × 0.53 ± 0.02 µm (width) at impeller speed of 600 rpm (Table 9). Cells abnormality did not appear in the culture at any impeller speed so far studied. Integrity and morphology of bacteria is sustained by the cell wall as imaged by SEM, which revealed the smooth surfaces in all impeller speeds tested in this study (Figure 8).

## 4. Discussion

Fermentation technology is widely used for the production of various economically important primary or secondary metabolites products. High productivity titer is the prerequisite for the industrial production of any type of metabolite. To optimise the metabolite yield, an optimisation of the production medium is required as the medium optimisation is one of the most widely studied processes conducted before any large-scale production of the metabolites. With the advent of modern mathematical and statistical techniques, medium optimisation has become more vibrant, accurate, reliable, economical and robust [12].

*L. lactis* is grown only in complex media and is therefore considered fastidious in nutrient requirements. Type of nutrients and their concentrations in the medium play an important role in commencing the maximum production of the metabolites as a limited supply of an essential nutrient may restricts the growth of microbial cells or product formation [12]. In this study, the OFAT experiments were applied to evaluate the influenced of medium components on BLIS production. In this classical medium optimisation technique, only one factor or variable is varied at a time while keeping other variables constant [21]. Because of its ease and convenience, the OFAT has been the most preferred choice among the researchers for designing the medium composition and this technique has been used in the initial stages in diverse fields [26]. OFAT can serve the purpose of rough approximation of the optimum levels [15]. The applications of OFAT in the assessment of influencing fermentation factors in the production of bacteriocin have been reported [27,28,29].

In OFAT experiments, the maximum BLIS production was recorded with the highest concentration of soytone (53.07 g/L). The nitrogen source in the soytone contains naturally occurring high concentrations of vitamins (magnesium, potassium, sodium, chloride, sulphate, phosphate), free amino acids (alanine, arginine, asparagine, aspartic acid) [30] and also carbohydrates of soybean [31]. The functions of amino acid for achieving high biomass of *Lactococcus lactis* IL1403 have been documented [32]. The free amino acids and growth factors in organic nitrogen sources contributed to the stimulatory effect on the production of bacteriocin-inhibitory compounds by *Lactobacillus plantarum* I-UL4 [33]. It is likely that the variety of nutrients present in organic nitrogen sources contributed to the stimulatory effect on BLIS production by *L. lactis* Gh1. Additionally, the results obtained in this study indicated that increase in soytone concentrations in the modified medium greatly enhanced BLIS production by *L. lactis* Gh1. A similar finding was reported by Ooi et al. [33] whereby production of bacteriocin-inhibitory compounds by *Lactobacillus plantarum* I-UL4 was increased linearly with the concentration of nitrogen sources. Similarly, Aasen et al. [34] and Khay et al. [35] also stated the beneficial of high nitrogen sources for the bacteriocins production by *Lactobacillus sakei* CCUG 42687 and *Enterococcus durans* E204, respectively.

Bacteriocin production by *L. lactis* Gh1 was also enhanced with increasing concentration of NaCl. Similarly, Venigalla et al. [14] also reported that bacteriocin production by *Lactobacillus plantarum* JX183220 was increased with increasing NaCl concentration and the highest activity was observed at 2.5 g/L NaCl. Besides its role for cell built-up, salt also plays a vital role in bacteriocin production as Na^+^ is essential to the osmotic pressure to the cells [36].

The conventional OFAT optimisation methods are the most common methods for improving fermentation medium components. However, OFAT is time-consuming, does not investigate the overall interaction between variables, is expensive especially for conducting a large number of experiments [21] and it may provide inaccurate data [22]. The statistical and mathematical approaches are practical and might overcome these limitations by changing more than one factor at a time [37]. Many studies claim that significant improvements have been made to the medium formulation obtained using OFAT techniques prior to the practise of design of experimental methods. Medium optimisation strategy using RSM or/and ANN methodology has been reported for enhanced production of bacteriocin from various LAB such as in *Lactococcus lactis* subsp. *lactis* [38], *P. acidilactici* Kp10 [39], *Streptococcus macedonicus* ACA-DC 198 [40], *Lactobacillus paracasei* J23 [41] and *Lactobacillus plantarum* ATM11 [42].

The statistical approaches of RSM and ANN are sequential strategies to design, analyse and find the optimum level and assessing the interrelationship effects of factors leading to the higher BLIS production by *L. lactis* Gh1. In RSM, researchers are allowed to design experiments and analyse the interactions between variables and responses during the entire study [22]. RSM is effective and a suitable design model that explains the combined effect and study several factors affecting fermentation responses by varying them in a limited number of experiments [43]. ANN, on the other hand, has recently emerged as one of the most efficient methods for empirical modelling and prediction in solving complex systems such as bacteriocin production. ANN is a highly simplified model mimicking the structure of a biological network. A set of biological neurons receive inputs, combine them, presents them as a nonlinear operation on the result and then output the final result [44,45]. ANN does not require prior specification of a suitable fitting function. ANN has the universal approximation capability, which means that it can approximate almost all types of nonlinear functions, including quadratic functions. The ability of ANN to predict the process characteristics with little prior knowledge is desirable, which simplifies their implementation and increases their modelling potential. This property makes ANN a powerful and flexible tool that is well-suited for modelling biochemical processes [46]. The RSM and ANN usually analyse a data set gained from the same experimental design, then both models are compared for their predictive capacity [22].

The finding of this study was in line with advantages offered by ANN over RSM, in which the prediction by ANN models was far more superior as compared to RSM using the same experimental design [22,38]. BLIS production by *P. acidilactici* Kp10 was about six times higher than that obtained in nonoptimized fermentation by incorporation of ANN in the optimisation of medium [18].

The effect of mixing in stirred tank bioreactor is an important environmental factor that affects growth performance or probiotic microorganisms. In addition to the uniform distribution of nutrients and heat in the bioreactor, good mixing is also necessary to prevent cells from being subjected to fluctuations in pH due to the intermittent action of pH control. Agitation is required to improve oxygen supply to the culture during the cultivation in stirred tank bioreactor. However, agitation is also related to shear rate effect. The degree of agitation has several effects on microbial growth which include cell wall disruption, changes in growth morphology, variations in the rates of growth and rates of formation of the desired product [47].

The impeller speed greatly influenced the production of BLIS by *L. lactis* Gh1. Results from this study demonstrated that the maximum BLIS activity (P_mX_) was recorded at 400 rpm, while the lowest BLIS production was recorded at the highest impeller speed (800 rpm). The finding of this study was in agreement with the study conducted by Abbasiliasi et al. [18] who stated that the production of bacteriocin from *P. acidilactici* Kp10 was only increased up to 400 rpm and significantly reduced at the agitation of above 500 rpm. Disadvantages of high impeller speed for bacteriocin production by LAB have been reported [48,49,50]. Reduced bacteriocin activity with increasing degree of agitation could be due to chemical degradation and effects on gene expression [5]. Moreover, in this study higher impeller speed was advantageous to cell growth but not optimal for BLIS accumulation. The biomass concentration for fermentation at 400 rpm was lower than that obtained at 800 rpm but BLIS concentration at 400 rpm was higher than detected at 800 rpm. Consequently, higher biomass concentration might not necessarily result in higher BLIS production. Several researchers have reported that the maximization of cell growth might not result in maximization of bacteriocin production [51,52,53]. Therefore, it was possible to obtain an optimal medium by optimizing the components of fermentation medium with BLIS production as the desired response.

It is interesting to note that low viability of *L. lactis* Gh1 cells was observed at high DOT level. Oxygen contributed the toxic effects on *Lactococcus lactis* by inhibiting its growth and survival [54,55]. Extended aeration of lactococcal cultures can cause DNA alteration and cell death due to the formation of hydroxyl radicals and hydrogen peroxide that may be the cause of the oxygen toxicity [56]. In this study, oxygen was not supplied throughout the fermentation; instead, oxygen was supplied only at the beginning of fermentation before inoculation until DOT level reached a maximum for the calibration of DO probe as 100% saturation. Then, the aeration was stopped to create an optional condition for the fermentation to progress on an optional basis.

Low bacteriocin activity is often a bottleneck in large-scale industrial production of bacteriocin. In the present study, BLIS production in 2 L stirred tank bioreactor was slightly improved as compared to fermentation in shake flask with not much variation in the maximum cell concentration and specific growth rate. Reduction of the production cost is the key factor for the economic viability of industrial production of bacteriocin for various food applications [57,58]. Improvement of yield is an important step in the scaling up of any fermentation product. Optimization of medium formulation is one of the key factors that need to be considered in the enhancement of any fermentation processes. Medium formulation for industrial scale fermentations should fulfil a number of criteria: it should be cost-effective, have high product yield and short fermentation time and exhibit ease of downstream purification processes [59].

## 5. Conclusions

RSM and ANN models were employed to optimize the medium formulation containing fructose, soytone, NaCl and Na_2_HPO_4_ for production of BLIS by *L. lactis* Gh1. Even though RSM can be used for the optimization of fermentation medium, ANN methodology provided better estimation point and data fitting as compared to RSM, with higher value of R^2^ and lower value of MAE and RMSE. BLIS production by *L. lactis* Gh1 in optimized medium consisted of 35.38 g/L soytone, 16 g/L fructose, 3.25 g/L NaCl and 5.40 g/L Na_2_HPO_4_ (717.13 ± 0.76 AU/mL) was about 1.40-fold higher than that obtained in nonoptimized medium (520.56 ± 3.37 AU/mL). BLIS production in 2 L stirred tank bioreactor was improved by about 1.18 times higher as compared to that obtained in 250 mL shake flask using the optimised medium, FST medium. The ability of ANN to predict process features with little prior information is beneficial, which simplifies their implementation and enhances the scope for modelling. This property makes ANN a powerful versatile method that is well suited to complex bioprocess modelling.

## Figures and Tables

**Table 1:** Selection of suitable concentration of modified Brain Heart Infusion (BHI) medium composition.

Commercial BHI Medium		Modified Medium	
Component	Concentration (g/L)	Component	Concentration (g/L)
Nutrient substrate (Extract of brain and heart, and peptones)	27.5 (4.6 g N)	Soytone	17.69 (2.3 g N), 35.38 (4.6 g N), 53.07 (6.9 g N)
Sodium chloride	5.0	Sodium chloride	2.5, 5.0, 7.5
Di-sodium hydrogen phosphate	2.5	Di-sodium hydrogen phosphate	1.5, 3.0, 4.5
Glucose	2.0	Fructose	1.0, 2.0, 4.0

**Table 2:** Experimental design of response surface methodology (RSM) and artificial neural network (ANN) independent variables on experimental and predicted bacteriocin-like inhibitory substances (BLIS) optimization by *L. lactis* Gh1.

Exp No.	Fructose(X_1_) g/L	Soytone(X_2_) g/L	NaCl(X_3_) g/L	Na_2_HPO_4_(X_4_) g/L	Bacteriocins Activity (AU/mL)			Final pH	Dry Cell Weight g/L
					Experimental	Predicted by RSM (% Difference) *	Predicted by ANN (% Difference) *		
1	4	38.46	1	1.5	598.16	562.90 (5.89)	598.16 (0)	4.43	0.51
2	12	38.46	1	1.5	593.93	600.48 (−1.10)	593.93 (0)	4.40	0.51
3	4	107.69	1	1.5	700.23	690.17 (1.44)	700.23 (0)	4.77	0.76
4	12	107.69	1	1.5	692.21	689.77 (0.35)	692.21 (0)	4.81	0.71
5	4	38.46	2.5	1.5	602.07	606.39 (−0.72)	602.07 (0)	4.39	0.50
6	12	38.46	2.5	1.5	626.86	617.78 (1.45)	626.86 (0)	4.38	0.51
7	4	107.69	2.5	1.5	676.64	703.61 (−3.99)	676.64 (0)	4.80	0.78
8	12	107.69	2.5	1.5	710.11	677.03 (4.66)	710.11 (0)	4.74	0.72
***9***	***4***	***38.46***	***1***	***4.5***	***589.26***	***600.60 (−1.92)***	***589.26 (0)***	***4.90***	***0.62***
10	12	38.46	1	4.5	658.06	622.02 (5.48)	658.06 (0)	4.46	0.62
11	4	107.69	1	4.5	663.64	663.65 (0)	663.64 (0)	5.02	0.92
12	12	107.69	1	4.5	673.15	647.09 (3.87)	673.15 (0)	4.72	0.87
13	4	38.46	2.5	4.5	644.18	637.54 (1.03)	644.18 (0)	4.84	0.63
14	12	38.46	2.5	4.5	644.44	632.76 (1.81)	644.44 (0)	4.44	0.63
15	4	107.69	2.5	4.5	698.83	670.54 (4.05)	698.83 (0)	4.96	0.91
16	12	107.69	2.5	4.5	601.61	627.79 (−4.35)	601.61 (0)	4.70	1.37
17	0	73.08	1.75	3	615.52	618.92 (−0.55)	615.52 (0)	5.41	0.74
18	16	73.08	1.75	3	586.34	613.75 (−4.67)	586.34 (0)	4.58	1.07
***19***	***8***	***3.85***	***1.75***	***3***	***530.23***	***553.07 (−4.31)***	***530.23 (0)***	***5.53***	***0.08***
***20***	***8***	***142.31***	***1.75***	***3***	***667.38***	***675.36 (−1.20)***	***667.38 (0)***	***4.90***	***1.45***
21	8	73.08	0.25	3	644.84	675.40 (−4.74)	644.84 (0)	4.60	1.15
22	8	73.08	3.25	3	699.34	699.59 (−0.04)	699.34 (0)	4.52	1.12
23	8	73.08	1.75	0	639.61	650.25 (−1.66)	639.61 (0)	4.56	1.00
24	8	73.08	1.75	6	618.54	638.71 (−3.26)	618.54 (0)	4.64	1.22
***25***	***8***	***73.08***	***1.75***	***3***	***611.25***	***608.99 (0.37)***	***608.99 (0.37)***	***4.58***	***1.11***
***26***	***8***	***73.08***	***1.75***	***3***	***585.35***	***608.99 (−4.04)***	***608.99 (−4.04)***	***4.63***	***1.18***
***27***	***8***	***73.08***	***1.75***	***3***	***599.66***	***608.99 (−1.56)***	***608.99 (−1.56)***	***4.61***	***1.14***
28	8	73.08	1.75	3	624.06	608.99 (2.41)	608.99 (2.41)	4.56	1.12
29	8	73.08	1.75	3	609.59	608.99 (0.10)	608.99 (0.10)	4.62	1.20
30	8	73.08	1.75	3	624.01	608.99 (2.41)	608.99 (2.41)	4.57	1.10

Note: The bold, and italic values (Exp no. 9, 19, 20, 25, 26 and 27) represent the experiments used for learning, and testing, respectively, by the selected ANN. * Percentages difference was calculated as the % difference between the observed value and corresponding predicted value over the observed value.

**Table 3:** The geometrical ratio of 2 L stirred tank bioreactor.

Parameters	Measurement
High of liquid (H_L_)	8.5 cm (for 1 L volume)
High of bioreactor (H_t_)	27.4 cm
Diameter of tank (D_t_)	13 cm
Diameter of impeller (D_a_)	52.96 mm
Diameter of baffles (D_b_)	9.94 m
Impeller blade height (W)	10.55 mm
Impeller blade width (L)	13.90 mm
Number of impellers	2
Impeller type	Ruston turbine

**Table 4:** Growth of *L. lactis* Gh1 and BLIS production in modified BHI medium at different media ingredients concentrations.

Ingredients (g/L)		Time P_mX_ (h)	pH		Maximum BLIS Activity P_mX_ (AU/mL)	Maximum Cell X_mX_ (g/L)	Specific Growth Rate μ_max_ (h^−1^)
			Initial	Final			
BHI		2	7.10	6.28	462.88 ± 1.08 ^f^	0.28 ± 0.004 ^f^	0.07 ± 0.001 ^j^
Fructose	1	6	6.71	5.29	542.95 ± 1.14 ^e^	0.50 ± 0.002 ^d^	0.12 ± 0.000 ^f^
	2	6	6.72	4.94	567.28 ± 5.78 ^d^	0.52 ± 0.004 ^cd^	0.11 ± 0.002 ^g^
	4	8	6.70	4.45	595.70 ± 6.75 ^b^	0.54 ± 0.004 ^bc^	0.13 ± 0.001 ^e^
Soytone	17.69	8	6.85	4.86	542.16 ± 2.76 ^e^	0.32 ± 0.002 ^e^	0.09 ± 0.000 ^h^
	35.38	6	6.77	4.89	583.69 ± 5.83 ^c^	0.53 ± 0.047 ^cd^	0.15 ± 0.002 ^c^
	53.07	6	6.69	4.94	620.35 ± 1.19 ^a^	0.69 ± 0.005 ^a^	0.16 ± 0.005 ^b^
NaCl	2.5	6	6.83	4.97	603.62 ± 5.41 ^b^	0.56 ± 0.005 ^b^	0.08 ± 0.002 ^i^
	5.0	8	6.77	4.89	557.50 ± 5.74 ^d^	0.56 ± 0.002 ^b^	0.12 ± 0.003 ^f^
	7.5	8	6.72	4.87	541.42 ± 4.40 ^e^	0.54 ± 0.002 ^b^c	0.14 ± 0.001 ^d^
Na_2_HPO_4_	1.5	8	6.67	4.67	559.13 ± 3.45 ^d^	0.52 ± 0.002 ^cd^	0.09 ± 0.000 ^h^
	3.0	8	6.79	5.04	560.75 ± 3.45 ^d^	0.56 ± 0.002 ^b^	0.12 ± 0.002 ^f^
	4.5	8	6.87	5.46	540.54 ± 6.82 ^d^	0.56 ± 0.004 ^b^	0.20 ± 0.001 ^a^
Ingredients					F = 0.380 (NS)	F = 0.156 (NS)	F = 0.713 (NS)
Concentrations					F = 0.147 (NS)	F = 1.457 (NS)	F = 6.82 (S *)
Interaction: Ingredients x concentrations					F = 94.12 (S **)	F = 81.84 (S **)	F = 273.46 (S **)

Note: All values are expressed as means ± standard deviation (SD) in triplicate. Data followed by the same letters are not significantly different (*p* = 0.05). Mean values of treatments were compared by the One-Way ANOVA followed by Duncan’s multiple range to evaluate the effect of investigated parameters. S: Significant; NS: Not significant; * Significant at *p* < 0.05; ** Significant at *p* < 0.001.

**Table 5:** Analysis of variance in the regression model for optimization of BLIS production by *L. lactis* Gh1.

Source	Sum of Squares	DF	Mean Square	F Value	Prob > F	
**Model**	**42,814.01**	**14**	**3058.14**	**4.17**	**0.0047**	**Significant**
A (Fructose)	40.04	1	40.04	0.055	0.8184	
**B (Soytone)**	**22,433.05**	**1**	**22,433.05**	**30.60**	**<0.0001**	**Significant**
C (NaCl)	877.33	1	877.33	1.20	0.2912	
D (Na_2_HPO_4_)	199.48	1	199.48	0.27	0.6096	
A2	92.63	1	92.63	0.13	0.7272	
B2	46.85	1	46.85	0.064	0.8039	
**C2**	**10,566.63**	**1**	**10,566.63**	**14.41**	**0.0018**	**Significant**
D2	2159.60	1	2159.60	2.95	0.1067	
AB	1441.91	1	1441.91	1.97	0.1812	
AC	685.88	1	685.88	0.94	0.3488	
AD	261.35	1	261.35	0.36	0.5594	
BC	902.62	1	902.62	1.23	0.2847	
**BD**	**4124.02**	**1**	**4124.02**	**5.63**	**0.0315**	**Significant**
CD	42.94	1	42.94	0.059	0.8120	
Residual	10,997.36	15	733.16			
Lack of Fit	9893.29	10	989.33	4.48	0.0558	Not significant
Pure Error	1104.07	5	220.81			
Cor Total	53,811.37	29				

**Table 6:** Comparison and validation including the predicted optimal value and BLIS activity obtained from the optimization of medium for BLIS production by *L. lactis* Gh1.

Fermentation Performance	Optimal Media Formulation (g/L)		Commercial BHI Medium (g/L)
	RSM	ANN	
Soytone	107.63	35.38	27.5
Fructose	4.0	16.0	2.0
NaCl	2.50	3.25	5.0
Na_2_HPO_4_	1.50	5.40	2.5
Predicted BLIS activity (AU/mL)	711.14	717.91	-
Actual BLIS activity (AU/mL) Verification experiment (% diff)	695.96 ± 2.48 (2.13)	717.13 ± 0.76 (0.1)	520.56 ± 3.37
MAE	15.44	2.20	
RMSE	27.08	26.02	
R^2^	0.79	0.98	

Note: MAE: mean absolute error; RMSE = root mean square error; R^2^ = coefficient of correlation determination.

**Table 7:** Growth of *L. lactis* Gh1 and BLIS production in BHI and FST media predicted by ANN in 2 L stirred tank bioreactor and shake flask.

Scale/Media	TimeP_mX_ (h)	Maximum BLIS Activity P_mX_ (AU/mL)	Maximum Cell Concentration X_mX_ (g/L)	Specific Growth Rate μ_mX_ (h^−1^)
Stirred tank bioreactor/Optimised (FST medium)	6	787.40 ± 1.30	0.87 ± 0.00	0.17
Stirred tank bioreactor/Unoptimized (BHI medium)	8	580.45 ± 19.79	0.43 ± 0.05	1.09
Shake flask/Optimised (FST medium)	12	665.28 ± 14.22	1.22 ± 0.061	0.10

**Table 8:** Kinetics of BLIS production by *L. lactis* Gh1 at different impeller speeds in 2 L stirred tank bioreactor.

Kinetic Parameter Value	Impeller Speed (rpm)				
	100	200	400	600	800
**BLIS production:**					
P_mX_ (AU/mL); maximum BLIS activity	772.87 ± 6.55 ^b^	787.40 ± 1.30 ^ab^	792.91 ± 3.90 ^a^	688.11 ± 12.34 ^c^	543.76 ± 6.83 ^d^
Y_BLIS/X_ (AU/g cells); cells productivity	790.53 ± 6.70 ^c^	966.21 ± 1.59 ^a^	837.58 ± 4.12 ^b^	694.65 ± 12.46 ^d^	523.02 ± 6.57 ^e^
q_p_ (AU/g/h); BLIS production rate	287.24 ± 7.98 ^a^	95.76 ± 7.43 ^ab^	200.76 ± 3.76 ^ab^	214.49 ± 65.72 ^ab^	17.74 ± 13.42 ^b^
**Cells:**					
X_mX_ (g/L); maximum dry cell weight	1.01 ± 0.004 ^b^	0.87 ± 0.002 ^d^	0.96 ± 0.005 ^c^	1.01 ± 0.002 ^b^	1.12 ± 0.002 ^a^
**Substrate consumption:**					
**1. Fructose:**					
S_mX_ (g/L); total fructose consumed	9.00 ± 0.14 ^b^	7.53 ± 0.04 ^e^	8.69 ± 0.13 ^c^	11.95 ± 0.07 ^a^	7.99 ± 0.01 ^d^
Y_BLISS/S_ (AU/g fructose); BLIS yield	87.12 ± 8.55 ^a^	72.60 ± 14.33 ^b^	87.54 ± 7.42 ^a^	70.17 ± 14.91 ^c^	59.38 ± 0.75 ^d^
Y_X/S_ (g cells/g fructose); cells mass yield	0.12 ± 0.00 ^b^	0.10 ± 0.00 ^e^	0.13 ± 0.00 ^a^	0.11 ± 0.00 ^d^	0.11 ± 0.00 ^c^
q_s_ (g/g/h); fructose consumption rate	0.34 ± 0.00 ^b^	0.23 ± 0.01 ^c^	0.09 ± 0.05 ^d^	0.58 ± 0.05 ^a^	0.33 ± 0.02 ^b^
**2. Nitrogen**					
S_mX_ (g/L); total nitrogen consumed	0.25 ± 0.07 ^ab^	0.15 ± 0.07 ^bc^	0.13 ± 0.04 ^bc^	0.08 ± 0.04 ^c^	0.35 ± 0.07 ^a^
Y_BLISS/S_ (AU/g nitrogen); BLIS yield	230.88 ± 1.94 ^b^	222.49 ± 0.38 ^bc^	251.34 ± 0.83 ^a^	208.58 ± 13.60 ^c^	178.28 ± 2.24 ^d^
Y_X/S_ (g cells/g nitrogen); cells mass yield	0.29 ± 0.00 ^c^	0.24 ± 0.00 ^d^	0.31 ± 0.00 ^b^	0.30 ± 0.00 ^b^	0.36 ± 0.00 ^a^
q_s_ (g/g/h); nitrogen consumption rate	0.03 ± 0.04 ^a^	0.03 ± 0.04 ^a^	0.03 ± 0.04 ^a^	0.03 ± 0.04 ^a^	0.02 ± 0.03 ^a^
**Production of lactic acid**					
LA_mX_ (g/L); lactic acid formed	2.44 ± 0.02 ^ab^	2.13 ± 0.11 ^c^	2.30 ± 0.07 ^bc^	2.49 ± 0.02 ^ab^	2.59 ± 0.15 ^a^
**Growth**					
µ_mX_ (h^−1^)	0.34 ± 0.008 ^a^	0.17 ± 0.002 ^d^	0.29 ± 0.002 ^c^	0.30 ± 0.001 ^b^	0.29 ± 0.001 ^c^

Note: Mean values with the different letters are significantly different according to Duncan’s multiple range test.

**Table 9:** The size of *L. lactis* Gh1 cells at various impeller speeds after 18 h of fermentation in 2 L stirred tank bioreactor.

Agitation Speed (rpm)	Size (µm)	
	Length (±SD)	Width (±SD)
Control (fresh cells)	1.10 ± 0.09 ^ab^	0.66 ± 0.04 ^a^
100	0.91 ± 0.07 ^c^	0.54 ± 0.06 ^bc^
200	1.06 ± 0.10 ^bc^	0.58 ± 0.03 ^b^
400	1.08 ± 0.20 ^bc^	0.51 ± 0.01 ^c^
600	1.26 ± 0.05 ^a^	0.53 ± 0.02 ^bc^
800	1.09 ± 0.06 ^ab^	0.53 ± 0.01 ^bc^

Note: All values are expressed as means ± standard deviation (SD) in triplicate. Data followed by the same letters are not significantly different (*p* = 0.05) according to Duncan’s multiple range test to evaluate the effect of investigated parameters.

## Data Availability

The data presented in this study are available on request from the corresponding author.
